# Single Nucleotide Polymorphism Network: A Combinatorial Paradigm for Risk Prediction

**DOI:** 10.1371/journal.pone.0074067

**Published:** 2013-09-11

**Authors:** Puspita Das Roy, Dhriti Sengupta, Anjan Kr Dasgupta, Sudip Kundu, Utpal Chaudhuri, Indranil Thakur, Pradipta Guha, Mousumi Majumder, Roshni Roy, Bidyut Roy

**Affiliations:** 1 Department of Biochemistry, University of Calcutta, Kolkata, West Bengal, India; 2 Department of Biophysics, University of Calcutta, Kolkata, West Bengal, India; 3 Institute of Haematology and Transfusion Medicine, Calcutta Medical College, Kolkata, West Bengal, India; 4 Department of Internal Medicine, Calcutta Medical College, Kolkata, West Bengal, India; 5 Human Genetics Unit, Indian Statistical Institute, Kolkata, West Bengal, India; University of Texas School of Public Health, United States of America

## Abstract

Risk prediction for a particular disease in a population through SNP genotyping exploits tests whose primary goal is to rank the SNPs on the basis of their disease association. This manuscript reveals a different approach of predicting the risk through network representation by using combined genotypic data (instead of a single allele/haplotype). The aim of this study is to classify diseased group and prediction of disease risk by identifying the responsible genotype. Genotypic combination is chosen from five independent loci present on platelet receptor genes *P2RY1* and *P2RY12*. Genotype-sets constructed from combinations of genotypes served as a network input, the network architecture constituting super-nodes (e.g., case and control) and nodes representing individuals, each individual is described by a set of genotypes containing M markers (M = number of SNP). The analysis becomes further enriched when we consider a set of networks derived from the parent network. By maintaining the super-nodes identical, each network is carrying an independent combination of M-1 markers taken from M markers. For each of the network, the ratio of case specific and control specific connections vary and the ratio of super-node specific connection shows variability. This method of network has also been applied in another case-control study which includes oral cancer, precancer and control individuals to check whether it improves presentation and interpretation of data. The analyses reveal a perfect segregation between super-nodes, only a fraction of mixed state being connected to both the super-nodes (i.e. common genotype set). This kind of approach is favorable for a population to classify whether an individual with a particular genotypic combination can be in a risk group to develop disease. In addition with that we can identify the most important polymorphism whose presence or absence in a population can make a large difference in the number of case and control individuals.

## Introduction

Acute Coronary Syndrome (ACS), otherwise known as myocardial infarction is one of the leading causes of increasing mortality rate in India. This infarction results from pathological thrombus formation and vascular occlusion in the coronary artery. In cardiovascular diseases, abnormal clotting occurs that can result in heart attacks. Blood vessels injured by smoking, cholesterol, or high blood pressure develop cholesterol-rich plaques that line the vessels [1]; these plaques can rupture and present sites for unwanted platelet binding. Following this, events occur which lead to formation of abnormal thrombus which blocks an intact blood vessel. Platelets are specialized disk-shaped cells in the bloodstream that are involved in the formation of blood clots. These clots are mainly responsible for causing heart attacks, strokes, and peripheral vascular disease. Therefore, platelets and ACS are present in a close proximity in cause and effect pattern [2].

One of the important events in this mechanism is the positive feedback cycle, initiated when platelets release several secondary mediators, such as Adenosine-di-phosphate (ADP), serotonin, etc. These agonists lead to activation and further aggregation of platelets which release more ADP. Abnormalities in ADP receptors, which are mediators in the above cycle, can predispose individuals to the formation of abnormal thrombus and hence ACS results [3]. Inherited abnormalities in components of the feedback cycle predispose individuals to develop the disease. The components found to play a major role in such pathology are the ADP receptor genes, *P2RY1* and *P2RY12* [4].

We have used these ADP receptor genes for our study with ACS patients and controls because platelets play one of the most important roles in thrombosis [5,6]. *P2RY1* and *P2RY12* play a pivotal role in platelet aggregation and there is a high degree of inter-individual variability in the platelet response to all agonists, in particular to ADP [7]. More importantly, this variation is reproducible over time in any given individual which points to a potential genetic influence on the platelet response to ADP [8,9]. The patient-wise variation of drug response can be traced back to polymorphism in *P2RY1* and *P2RY12* among other factors [10].

**Figure 1:**
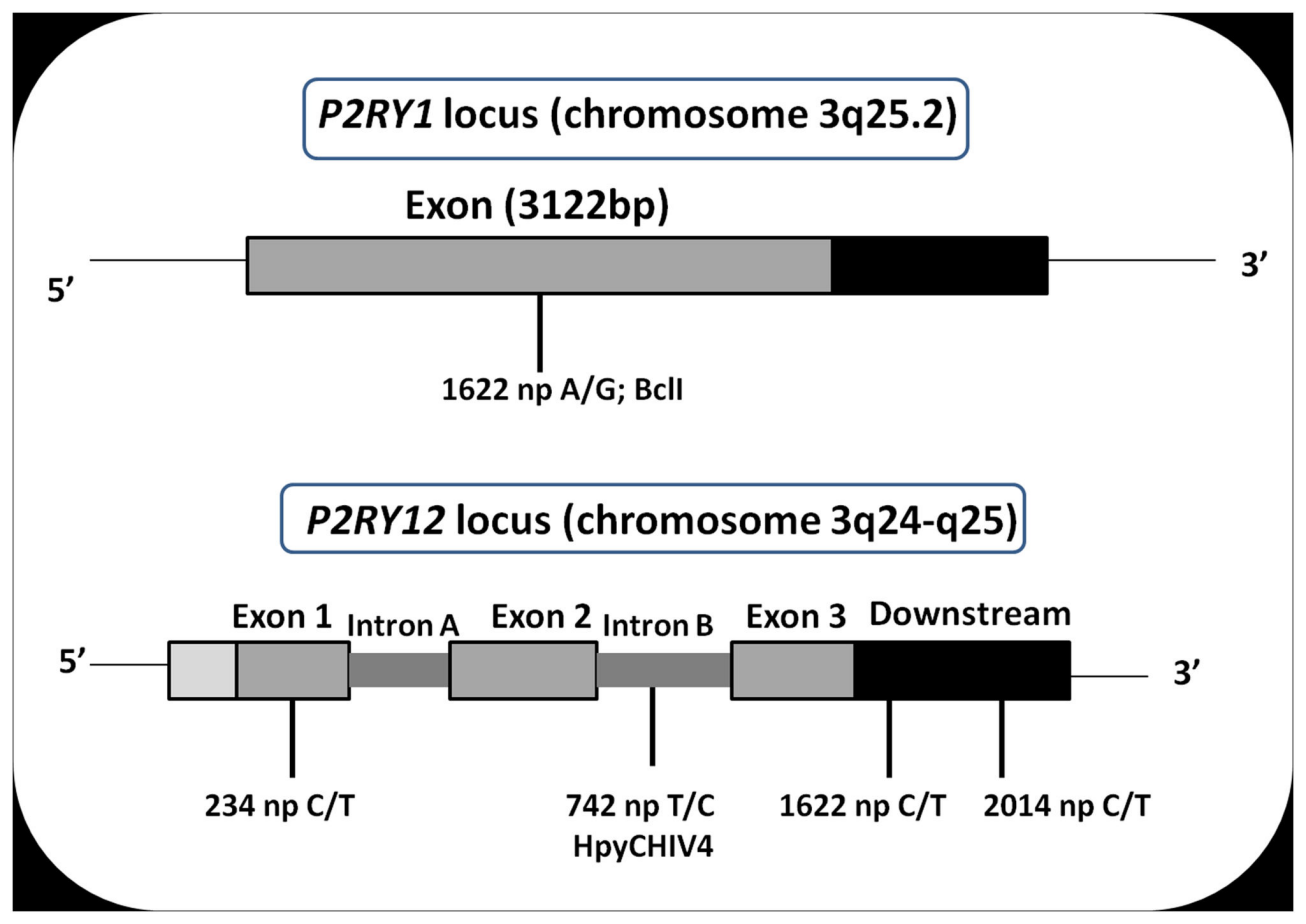
Single nucleotide polymorphic positions at *P2RY1* and *P2RY12.* Polymorphic and corresponding restriction enzyme cutting sites at *P2RY1* and P2RY12 (*Distance between two sites is not in proper scale*).

*P2RY1* and *P2RY12* genes are located on chromosome 3 (Figure 1). The *P2RY1* gene spans 4 kb and is made up of a single exon of 3122 base pairs encoding a 372-aa protein [11]. The *P2RY12* genes spans 47 kb and is made up of 3 exons and 2 introns. Hollopeter et al. [12] found platelets to express the shorter transcript variant, consisting of sequence from exons 2 and 3. The entire coding sequence for the 342-aa receptor is found in exon 3. Rare mutations within the *P2RY1* 2 gene have been shown to disrupt receptor function and lead to a bleeding diatheses [13]. Whether there are more common variants in the two genes, which influence inter-individual variation in platelet reactivity to ADP, has only recently come under investigation [14]. Several studies have been performed to identify the responsible polymorphisms present on the platelet receptor genes, associated with the different patterns of platelet aggregation or dose dependency of aspirin/ clopidogrel in several populations across the world. However, in the eastern Indian population, where ACS is a major cause of death, no such studies have been performed to identify the risk genotype and to trace the cause behind inter-individual variation.

After getting the responsible SNP by doing conventional statistical analysis we have tried to introduce a network based on combined-genotypes of different loci to develop a genotype risk index in the sampling population. This new method also provides a graphical visualization tool to inspect the strengths of different genotypic sets (from network edges) suspected to be correlated with either (i) presence or (ii) absence of a disease. Moreover, the most important genotype can be identified through the network approach. This has been modelled using experimentally generated genotypic data sets from acute coronary syndrome patients and control individuals on the basis of the genotypes present at five different loci on platelet receptor genes *P2RY1* and *P2RY12*. Importantly, this approach towards case-control studies might be equally applicable in other disease associated genotypes based prediction systems and to verify it we have also used a moderately large dataset of oral cancer, pre-cancer and control individuals from the same population.

## Materials and Methods

**Figure 2:**
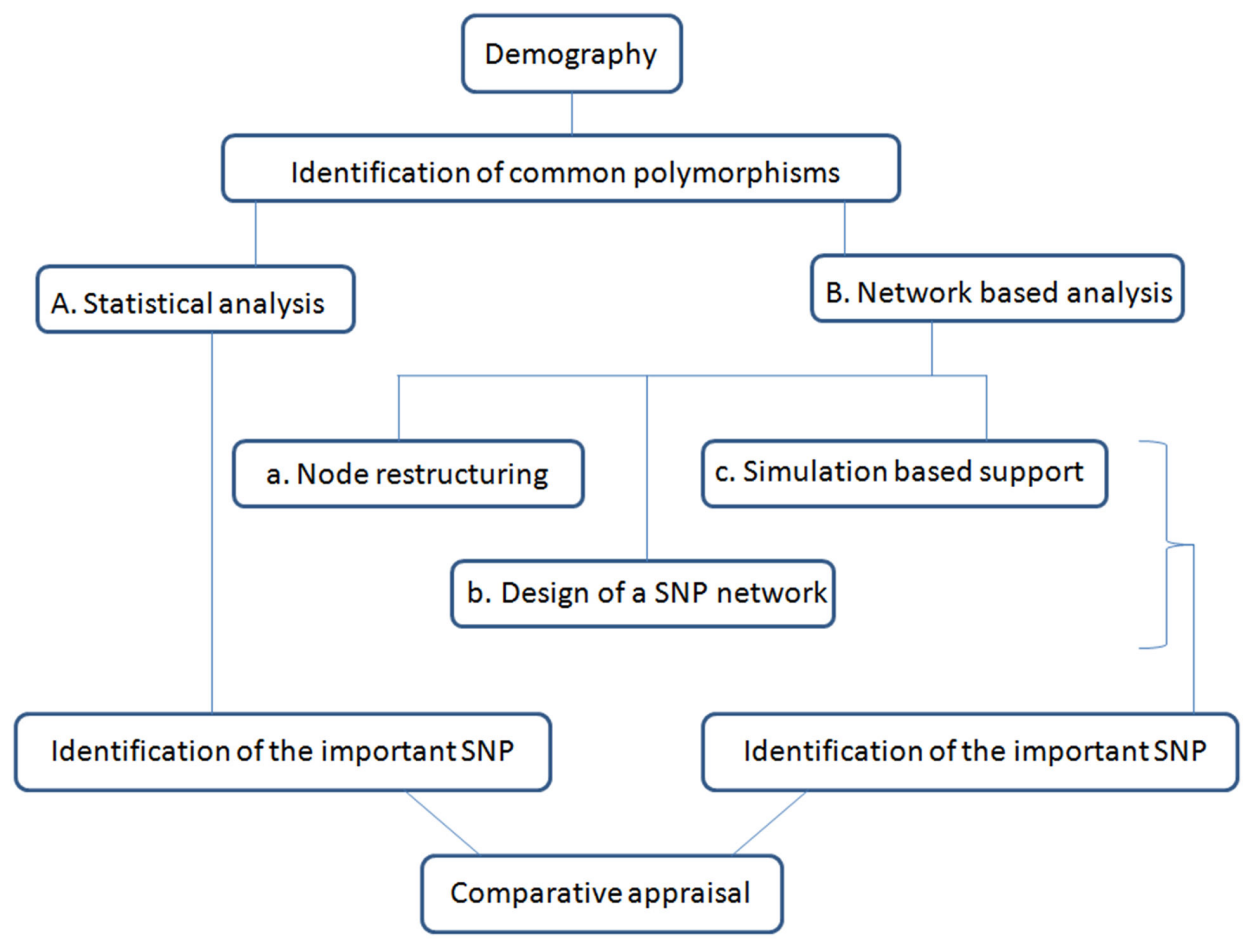
The strategy of our work is described by the illustration. The chart shows the strategy employed in the present analysis. The way of doing the whole analyses is described sequentially through the chart. The methods involved in the network based analysis and further the consistency of the outcome of Network based approach and the conventional statistical methods are also described.

The strategy of the whole work is described through Figure 2.

### Subjects and sample collection

A total of 177 blood samples were collected from both patients with acute coronary syndrome [ACS] (n=91) and non-ACS (n=86) individuals. Ethnicity and particular age bar was maintained during blood collection. Patients with ACS were under the medication of anti-platelet drugs for 4-5 days and the non-ACS individuals were without any bleeding disorder. Family history of disease was collected from cases and controls along with their disease -history.

### Ethics Statement

The study was approved by the Bio Ethics Committee for Animal and Human Research Studies, University of Calcutta and all subjects gave written informed consent.

About 500µl blood from ACS patients was collected in EDTA vials after 4-5 days of their admission and with proper medication. Similarly, from non-ACS individuals also 500µl blood was collected in EDTA vial. All subjects were rested supine for at least 20 minutes before venepuncture to minimize the effects of stress hormones, and blood was collected using a standardized phlebotomy technique designed to minimize platelet activation [15]. Genomic DNA was isolated from each blood sample by using QIAGEN blood DNA isolation kit.

### *P2RY1* and *P2RY12* Gene Screening for Common Polymorphisms of ACS

Each gene was screened by PCR using sets of five overlapping primer pairs each amplifying segments of ~1kb in length. PCR was performed on Applied Biosystem Thermal Cycler using a proof-reading DNA polymerase (Fermentas).

#### Genotyping at P2RY1 and P2RY12 polymorphisms

Four polymorphisms were typed at *P2RY12* and one at *P2RY1* (Table S1 in File S1). Polymorphisms were chosen that would give the greatest power to determine an effect. In the *P2RY1*, we therefore selected the 1622*A*>*G* (SNP1) polymorphism. For the *P2RY12*, we selected *IntB742T>C* (SNP2) (to represent the 5 linked polymorphisms), 234C>*T* (SNP3), 1622C>*T* (SNP4), and 2014C>*T* (SNP5) polymorphisms. The 1622*A*>*G* polymorphism at *P2RY1* and *IntB742T>C* polymorphism at *P2RY12* were detected by PCR amplification and allele specific restriction endonuclease digestion with BclI and *HpyCHIV* 4 (New England Biolabs, Herts, UK) respectively. The 234C>*T*, 1622C>*T* and 2014C>*T* polymorphisms at *P2RY12* were typed by re- sequencing the PCR products.

#### Detection of P2RY1 1622A>G and P2RY12 IntB742T>C polymorphism

**Figure 3:**
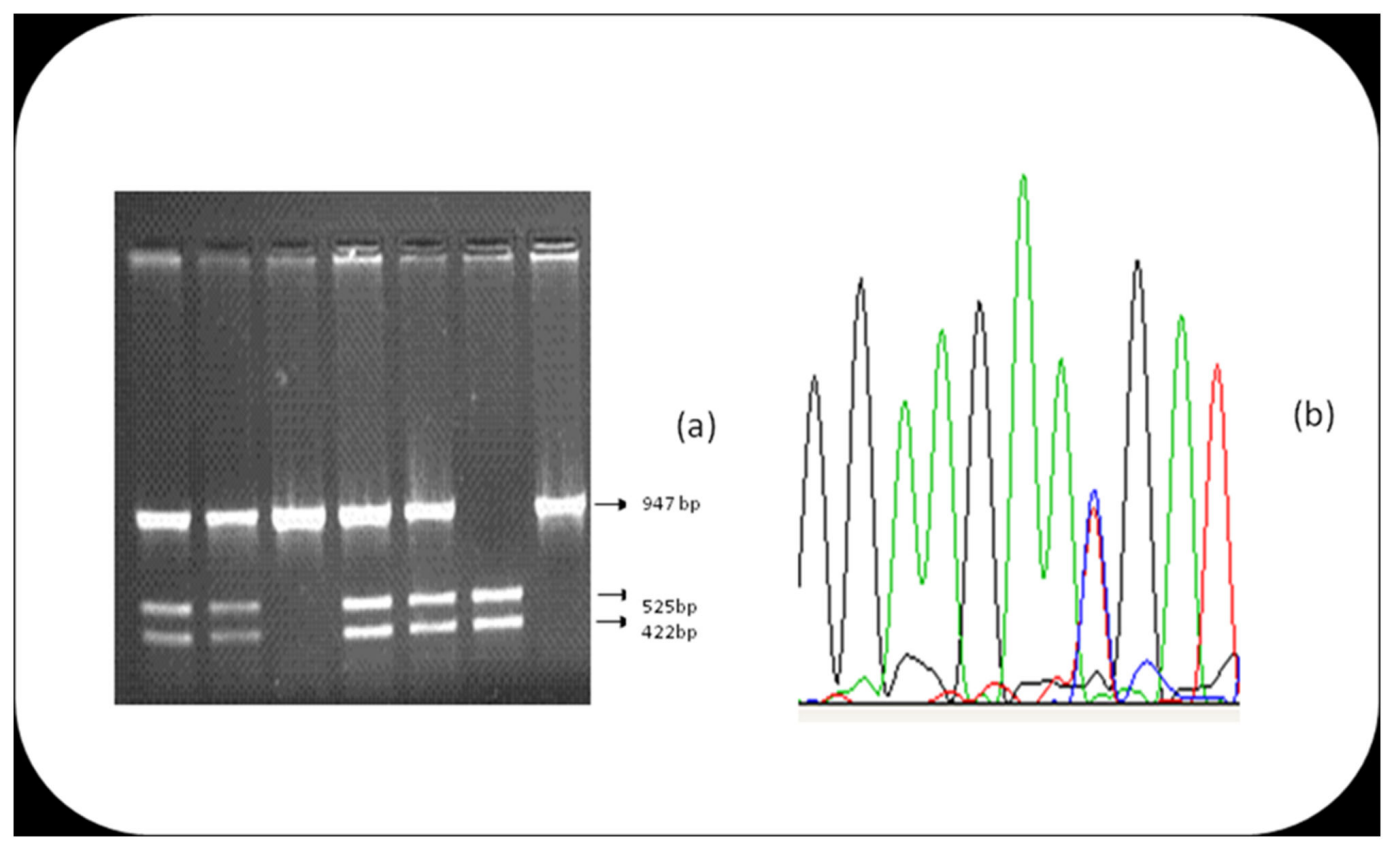
Agarose gel photograph and a chromatogram view of the 5 loci. (a) Agarose gel photograph of RFLP using Bcl1 enzyme on *P2RY1* gene (*1622A>G*). (b) Chromatogram view of sequencing of *P2RY12* (*1622T>C*).

Initially, part of the *P2RY1* and *P2RY12* gene was amplified by PCR using the specific primers (forward primer for *P2RY1* - 5’-GCCATGTGTAAACTGCAGAGGTTC-3’, reverse primer- 5’-CTTGTTTGGGTTTGCTTTCACAGT-3’ and forward primer for *P2RY12*- 5’-CATTTTGGGGAATTTAAGTGC-3’, reverse primer- 5’-GAGAGGATGGTTATTTTCAGCC-3’). The PCR was run for 35 cycles and the first cycle was preceded by an initial denaturation at 95^0^C for 5 min and the last cycle was followed by 3 min of final extension at 72^0^C. Each cycle consists of 1-min denaturation at 95^0^C, 2-min annealing at 57^0^C and 1.5-min elongation at 72^0^C. The reaction mixture for amplification included 1.0 or 2 mM MgCl_2_ (for *P2RY1* and *P2RY12*, respectively), 1 unit Taq DNA polymerase (Fermentas), 100–150 ng of DNA, 200 µM dNTPs and 2 pmol of primers. A portion of each reaction product was electrophoresed on 2% agarose gel to check for the desired products (947bp and 1146 bp respectively). If the amplified products were detected, then the remaining portions of the samples were digested with 5 Unit of BclI and *HpyCHIV* 4 (New England Biolabs, Herts, UK) respectively. The digested products were analyzed in 2.5% agarose gel to identify the genotypes (*AA/AA*-947bp, *AG/AA*-525bp, 422bp, 947bp, *GG/GG*- 525bp, 422bp for *P2RY1* (Figure 3) and *TT/TT*-744bp, 402bp, *TC/TT*-744bp, 580bp, 402bp, 164bp, *CC/CC*-580bp, 402bp, 164bp for *P2RY12*).

#### Resequencing of PCR products for genotyping of 234C>T, 1622C>T and 2014C>T polymorphisms in the P2RY12 gene

PCR reactions have been done using two sets of primers; one amplifies the region that contains 234C>*T* (forward primer 5’-TCTCTGATTGTGAAGCCCTC-3’ and reverse primer 5’-TGGCATCTACATCTTGGGAA-3’ and the other amplifies the region containing both 1622C>*T* and 2014C>*T* (Forward primer 5’ CAAACGACATCCAATTGTCA 3’ and reverse primer 5’ TGTATATGGTATGGTGAGTCATGG 3’). These PCR products were then subjected to resequencing (ABI, Foster City, CA, USA) by using forward primers and big dye terminator to detect the type of nucleotide present at the particular position (Figure 3).

### DNA repair gene screening for polymorphisms of oral cancer

We have genotyped five polymorphisms in the DNA repair genes [*OGG1* (SNP1), *XRCC1* (SNP2), *XRCC3* (SNP3), *XPC* (SNP4) and *XPD* (SNP5)] for oral cancer to identify the alleles present on these regions.

#### Oral cancer, leukoplakia (precancer) patients and controls recruitment for DNA isolation

Oral cancer (n=298), leukoplakia (n=219) patients and healthy controls (n=369) were recruited from R. Ahmed Dental College And Hospital (a primary referral hospital at Kolkata, capital of West Bengal, a state in Eastern India) during 1999 to 2006 and isolation of DNA were done as described in previous publication [16].

#### Polymorphisms at OGG1 (Codon 326, Cys/Ser)

All samples were screened for the polymorphism at codon 326 of *OGG1* (*Cys>Ser*). Polymorphism at this locus was determined by PCR in a 10 µl reaction volume containing 10 mM Tris-HCl (pH 8.3), 50 mM KCl, 1.0 mM MgCl_2_, 20 µM of dNTPs, 2 pmol each of the primers, 50^~^100 ng of genomic DNA and 0.5 U of Fast Start Taq DNA polymerase. The PCR product of 156 bp was digested with ItaI (New England Biolabs Inc, Beverly, MA, USA) and electrophoresed in 3% agarose gel. Banding patterns for genotypes were Ser/Ser =100, 56 bp, Ser/Cys=156, 100, 56 bp and Cys/Cys =156 bp [17].

#### Polymorphisms at XRCC1 (codon 399, Arg/Gln) and XPD (codon 312, Asp/Asn)

Genotypes at these SNP sites were determined as described previously [16].

#### Polymorphisms at XRCC3 (Codon 241, Thr/Met)

Polymorphism at codon 241 of *XRCC3* was determined by generating a 552bp PCR product, digesting with *Nla*III and electrophoresing in 2.5% agarose gel [18]. The 10 µl reaction mixture comprised of 10mM Tris-HCl (pH 8.0), 50mM KCl, 2mM MgCl_2_, 50^~^100 ng of template DNA, 3 pmol of each primer, 20 µM dNTPs and 0.5 U of Fast Start Taq DNA polymease. In addition to the polymorphic site at codon 241, one additional monomorphic *Nla*III site, producing a 239bp DNA fragment, served as an internal control for restriction enzyme digestion. Genotypes were determined by banding patterns such as: *Thr/Thr* (313,239 bp); *Thr/*Met (313,239,208,105 bp); and *Met/*Met (239,208,105 bp).

#### Polymorphisms at XPC (Codon 939, Lys/Gln)

Polymorphism at codon 939 of *XPC* was determined by digesting the 765bp PCR product with PvuII (New England Biolabs Inc, Beverly, MA, USA) for overnight at 37^o^C and electrophoresing in 1.5% agarose gel. A PCR reaction of 10 µl reaction volume containing 10 mM Tris-HCl (pH 8.3), 50 mM KCl, 1.25 mM MgCl_2_, 100 µM of dNTPs, 2 pmol each of the primers, 50^~^100 ng of genomic DNA and 0.5 U of Fast Start Taq DNA polymerase. Banding patterns were Lys/Lys=585,180bp, Lys/Gln=765,585,180 bp and Gln/Gln =765 bp [19].

### Statistical Analysis

Pearson chi-squared test with Yates’ correction was performed to determine whether the association between polymorphisms at each site and the disease is significant. Adjusted (age, sex) risk of ACS was calculated as odds ratios (ORs) with 95% confidence intervals (CIs) by binary logistic regression analysis using SPSS statistical package (version 10.0, SPSS Inc., Chicago, IL, USA). Pair wise linkage disequilibrium (LD) was calculated using an algorithm named as HAPLOVIEW (http://www.broad.mit.edu/mpg/haploview/). Frequencies of different alleles/haplotypes were estimated using genotype data by the maximum-likelihood method using the algorithm named as HAPLOPOP (http://www.docstoc.com/docs/68174433/HAPLOPOP).

### A network based genotype ranking

#### Network Architecture

**Figure 4:**
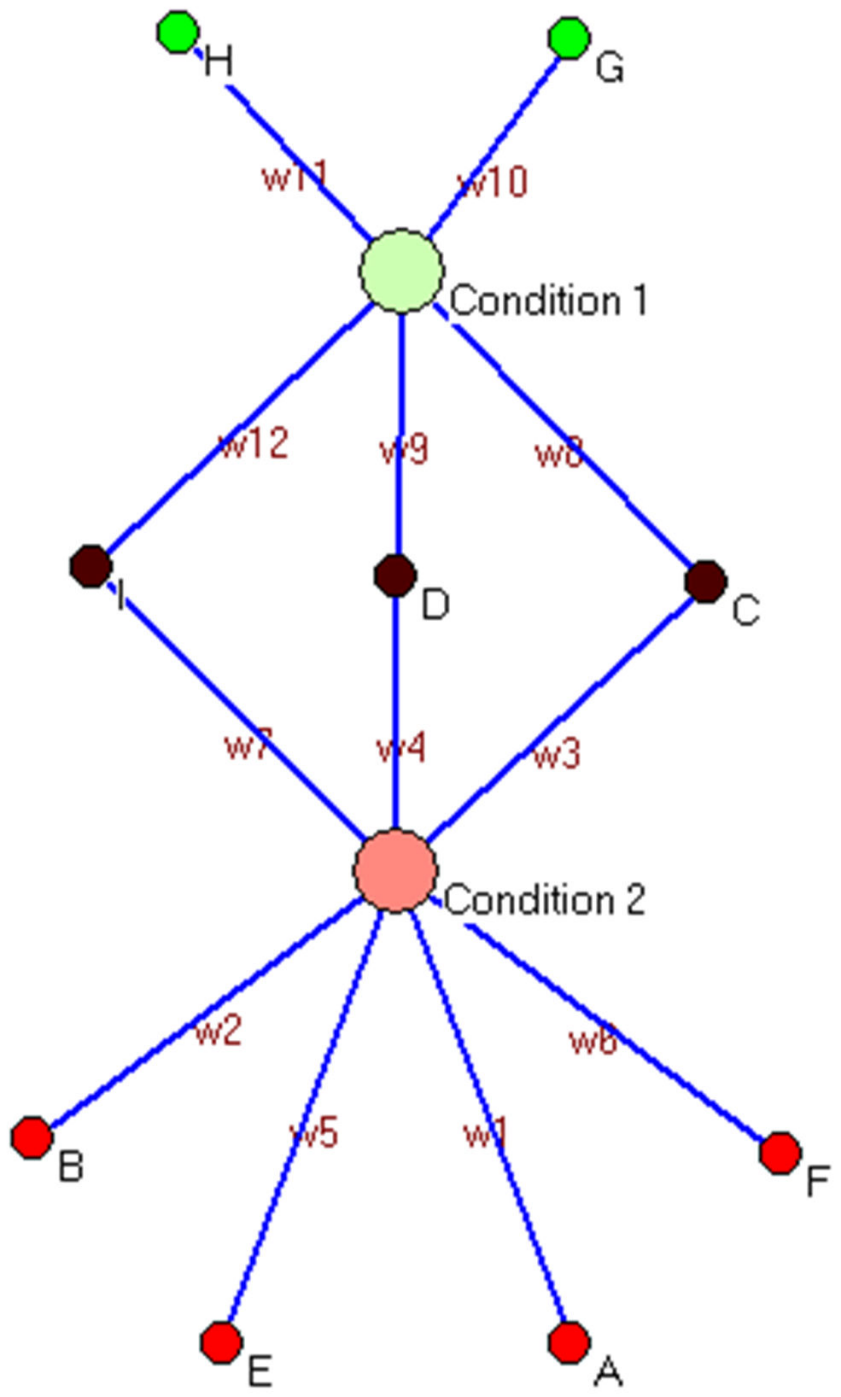
Toy network to understand the whole network architecture strategy. The toy network contains 2 supernodes (Condition1, Condition2), 8 nodes (from A-H) and w1-w12 edges through which nodes are linked to supernodes. In this network, Fraction of Condition2 specific genotypes =4/9 and Fraction of Condition2 specific population = (W1+W2+W5+W6)/∑ W.

Let us consider a network **R** in which we introduce **k** super-nodes, implying nodes with different stages of a given disease (e.g. ACS). Such stages can be binary in nature (like case and control, k=2) or can have multiple stages (e.g. control, precancer and cancer, k=3). While super-nodes represent different conditions, each of the nodes (**N**) represents combination of **M** markers; each marker may be a genotype. In the simplest possible case, the markers may be number of allele pairs, each allele representing a polymorphic site. Each node is connected at least one or maximally k super-nodes and the edge width ‘**w**’ satisfies 1 = < w< P (where P is the total sampling population). For better understanding, the above can be explained with the help of a toy network. Figure 4 represents a toy network where there are k=2 super nodes each representing different conditions (condition1 and condition2) and total number of nodes [N = 9 (A to I)] are representing unique combination of different markers connected to the two different conditions. The edge-labels w1 to w12 correspond to the number of individuals having a particular unique combination of markers. Therefore, from the network we can calculate the condition1 and/or condition2 specific genotype as well as genotype specific individuals.

For each polymorphism in this study, the alleles are designated by numerical digits '1' and '2' (wild and mutant respectively). For example, in case of SNP at position *P2RY1* 1622 A>G, a patient with genotype AA is referred as 11, AG or GA as 12 and GG as 22 in our study. Next, we combined the genotypes of all the 5 SNPs consecutively to form genotypic combination (super set genotypes) for each patient (eg. 12 22 11 22 11).

A node with M=5 with characters 12 12 22 11 12 would imply homozygosity in two positions and heterozygosity in the rest three. It should be noted that node 12 12 22 11 12 and the node with character 21 21 22 11 21 are equivalent, but in contrary the node 12 12 22 12 11 is non-equivalent with the node 12 12 22 11 12.

From this combined genotypic data, it became possible to generate a network scheme that can easily segregate the genotype-sets as different clusters which are case specific, control specific and some genotypic sets (common) which belong to both the super-nodes. These segregated groups can also be easily visualized using network based software Pajek [20]. The individuals with incomplete information at any one SNP were removed from this study.

The width of the edges clearly shows which particular genotype-set is occurring more frequently in cases and which in controls in the studied population. It can be possible from the network to predict individuals containing a particular genotype-set are inclined to which cluster. The scheme enables a clear segregation of genotype-sets with the referred attributes in the population (Tables S2.A and S2.B in File S1 are showing the segregation pattern for ACS-control and oral cancer-precancer-control, respectively).

**Table 1:** Distribution of genotypes in ACS patients and controls.

**Gene**	**Polymorphism**	**Genotype**	**Frequency in ACS n(%)**	**Frequency in control n(%)**	**p value**
P2RY1	1622 A>G	GG	3 (0.03)	2 (0.03)	0.07
	SNP1	AG	34 (0.39)	19 (0.25)	
		AA	51 (0.58)	56 (0.73)	
		A	68 (0.23)	12 (0.15)	0.02*
		G (Ref.)	20 (0.77)	65 (0.85)	
P2RY12	234 T>C	TT	21 (0.24)	17 (0.22)	0.06
	SNP2	CT	51 (0.58)	56 (0.73)	
		CC	16 (0.18)	4 (0.05)	
		T (Ref.)	46 (0.53)	45 (0.58)	0.5
		C	42 (0.47)	32 (0.42)	
	742 T>C	TT	4 (0.04)	6 (0.08)	0.26
	SNP3	CT	11 (0.12)	15 (0.19)	
		CC	73 (0.83)	56 (0.72)	
		T	9 (0.11)	14 (0.18)	0.21
		C (Ref.)	79 (0.89)	63 (0.82)	
	1622 C>T	CC	27 (0.31)	74 (0.96)	0.05
	SNP4	CT	51 (0.58)	2 (0.03)	
		TT	10 (0.11)	1 (0.01)	
		C (Ref.)	52 (0.60)	75 (0.97)	0.004*
		T	36 (0.40)	2 (0.03)	
	2014 C>T	CC	61 (0.69)	56 (0.73)	0.49
	SNP5	CT	25 (0.28)	20 (0.26)	
		TT	2 (0.02)	1 (0.01)	
		C(Ref.)	73 (0.84)	66 (0.86)	0.79
		T	15 (0.16)	11 (0.14)	

**SNP1**, G > A allele, OR= 2.1, 95% CI= 1.1-4.03; **SNP4**, C>T allele, OR=20.05, 95% CI= 1.0-399

significant p values are marked as ‘* ’

#### A combinatorial approach

Given the above mentioned architecture R for the network consisting of N nodes and k super-nodes we can now construct a network with M’ markers (with say, M’=M-1). Now, in the network R' with M’ markers, one can have N’ possible combinations each with a missing marker. The total number of unique combinations (N') differs with different marker combinations since once a particular marker is removed, two nodes (i.e. unique combinations) differing at that particular maker position can lose their variation and get collapsed to a single node (i.e. common combination).

For example, say there are three unique genotype combinations viz. 12 22 11 11 11, 12 22 11 11 12 and 12 22 11 11 22. Now when we study how the population restructures if the fifth SNP is not considered, the three unique genotypes collapse to a single genotype i.e. 12 22 11 11. The idea is to generate a network R' that is equivalent to the condition when one marker is absent in the study.

Different networks are generated after omission of each marker/ SNP. One can have different configurations of R' when one marker is omitted at a time. The marker whose absence affects the case–control ratio by changing the average degree of super-nodes emerges out to be the most decisive marker of the study.

Now, the frequency of nodes specific to case and control emerging as a result of omission can be counted for each network. As for example, Figure 4 contains fraction of condition2 specific nodes= 4/9 and fraction of condition2 specific individuals = (W1+W2+W5+W6)/∑W

### Simulation based support

#### Simulation with constant number of genotypes (Method 1)

For this given population (ACS-control), with a given number of SNPs, we started with shuffling the genotypes for each SNP across the entire population such that the number remains constant to see the consistency of our newly proposed method. For example, if we have 5 SNPs, the total number of homozygous dominant, homozygous recessive and heterozygous genotypes for each SNP remains constant (The genotypes are shuffled across the population, not across different SNPs). So, when the genotypic supersets are formed, they are basically permuted genotypes of the 5 SNPs across the population. This method is repeated 1000 times to generate 1000 random datasets (Type 1 dataset).

#### Simulation with constant number of alleles (Method2)

We have counted the allele frequency for this particular population (ACS-Control) for each SNP and shuffled the genotype such that the number of individual alleles per SNP remains constant, but its count of homozygous dominant, homozygous recessive and heterozygous genotype changes. The same method of shuffling is done for all the SNPs in data set. This method is repeated 1000 times to generate 1000 random datasets (Type 2 dataset).

In both the cases the node deletion study is performed and depending on the restructuring of case specific and control specific population, risk SNPs are ranked (depending upon the number of case specific genotype supersets remained after SNP removal from dataset or the ratio of case specific and control specific genotypic super sets) for each dataset. Finally, we calculated the number of times each SNP from the random dataset appears in the exact same rank as that of the original dataset. The normalized values for these two methods are presented in Tables 3 and 4.

#### SNP number optimization with large SNP set

With the aim to see the effect of our newly proposed approach on the large number of SNPs we have adapted the simulation method with a large case-control dataset of 1804 SNPs from 635 individuals (obtained from Dr. Ananyo Choudhury, University of Witwatersrand, Johannesburg, South Africa). About 5, 10, 15, 20, 25, 30, 50, 100, 150, 200, 250 numbers of SNPs (SNP subset) are randomly picked up. Genotype supersets are formed from these SNP subsets for both case and control populations. The numbers of case-specific control-specific and common genotype supersets are calculated for each SNP subset. The whole process is repeated for 1000 times to see the consistency of the result.

## Results

### Demography of retrospective study

All patients and controls were living in and around the city of Kolkata, located on the eastern region of India. Most of the patients and healthy controls belonged to low-income group (family income <100 US$ per month) and had similar nutritional status. None of the patients and controls was exposed to specific occupational or environmental stress so environmental effects were similar. Average age of both case group and control group was 56.8+2 years (range 25 to 70 years). In cases, a total of 83.52% were male and in controls, a total of 64% were male.

Demographic details and tobacco habits of oral cancer, precancer and control populations are described in previous publication [21].

**Table 2:** Haplotypes of two loci (P2RY12 742IntB T>C and P2RY12 1622C>T) which are likely to be linked (R^2^=0.7) and their corresponding odds ratio at 95% confidence interval and p-values.

**Haplotypes**	**Allelic designation of haplotypes**	**Total number in case (N=176)**	**Total number in control (N=154)**	**Odds ratio**	**95% Confidence Interval**	**p-value**
1--1	C-C	13	17	0.59	0.2722-1.2775	0.1238
2--1	T-C	102	135	0.19	0.1076-0.3336	0.0001*
2--2	T-T	61	2	39.31	9.413-164.1693	0.0001*

The haplotypes which are present in >3% in either case and/or control population are taken for risk assessment and we mark the significant p-values as *

**#n = number of individual, N = 2n**

### Conventional statistical analysis

All polymorphisms were in Hardy-Weinberg equilibrium in the control population. As evident from Table 1 that details the purinergic receptor linked polymorphisms in ACS, carrying *A* allele at *P2RY1* 1622 position and *T* allele at *P2RY12* 1622 position were significantly associated with the disease (p=0.02 [OR= 2.1, 95% CI= 1.1-4.03] and p=0.004 [OR=20.05, 95% CI= 1.0-399] after adjustment of age and sex). HAPLOVIEW software shows that the SNPs are not in complete linkage disequilibrium (LD), but the LD between *P2RY12* 742*T*>*C* and P2RY12 1622C>*T* gives a R^2^ value of 0.7. Therefore it can be said that the two SNPs are moderately linked. We considered those haplotypes for risk assessment which are >3% in either of the population of case and control. Table 2 shows the haplotypes and their corresponding p-values.

### Network based analysis

#### Node restructuring

**Figure 5:**
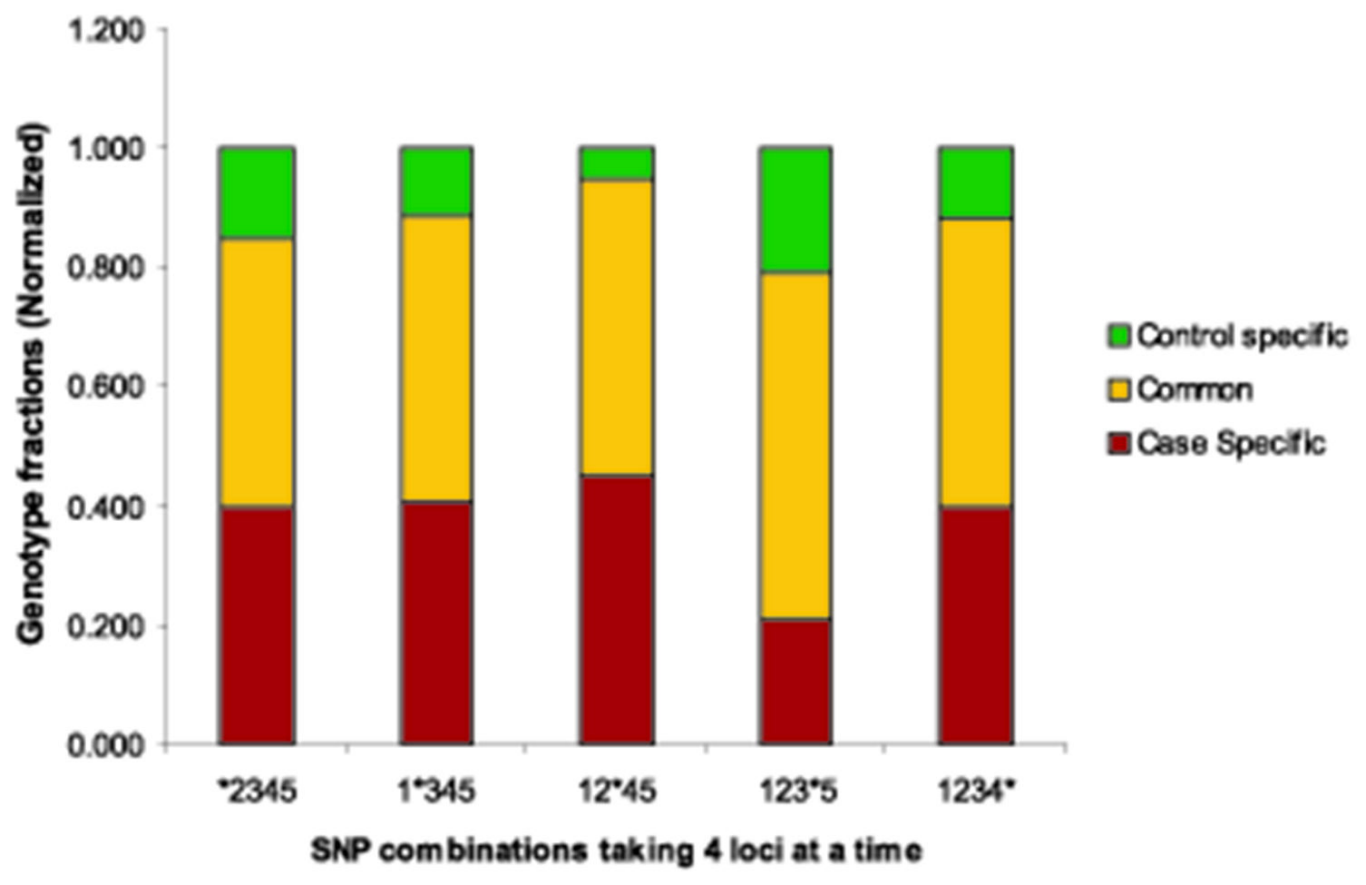
Case and control specific genotypic fractions after single locus omission. One SNP at a time is removed from all the genotypic set in the population to predict the probable risk genotype. The removed locus is denoted by * in the genotype supersets formed taking 4 loci at a time. The effect is studied in terms of the redistribution of number of unique genotypes (nodes) remaining after each SNP deletion in Case, Control and Common populations. The total number of genotype differs with different SNP combinations since once a particular SNP is removed; two genotypes may lose their variation and get collapsed to a single genotype.

**Figure 6:**
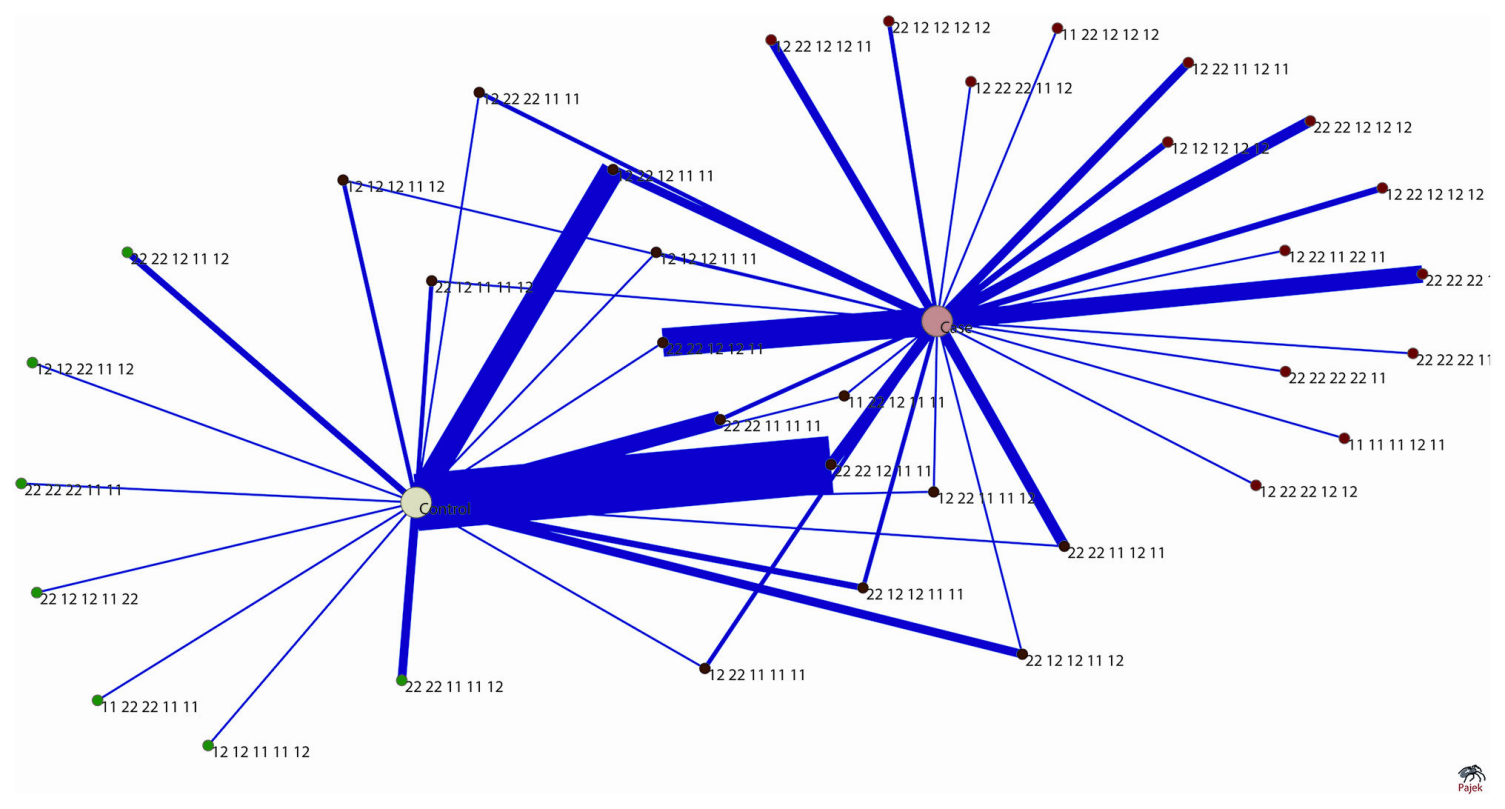
A network through which we represent the segregated pattern of combined genotypic data of case (acute coronary syndrome) and control population (healthy). The 5 SNPs of each individual are combined to form super-set genotypes in both case and control. Thirty five unique genotype combinations are observed of which 14 combinations are specific to cases (marked as red), 7 combinations are specific to controls (marked as green) and 14 combinations are present in both case and control (marked as brown). The number of occurrences of each particular genotype combinations is illustrated through its corresponding edge-width.

It would be interesting to find whether the genotypic supersets can be used to predict a risk locus and more importantly, whether it matches the conventionally statistically derived risk locus (as mentioned above). So, when one SNP was removed at a time one from the genotype superset (without altering their positions), the total number of unique genotypes (here 35) decreases since the omission of each SNP reduces some variability and consequently two or more node collapses to a single node. Now the fraction of nodes specific to case and controls emerging as a result of omission of one SNP, at a time, are counted. This situation is equivalent to the results that we would have obtained if that omitted SNP was not at all included in the study from the beginning. As a result of single SNP omission at a time, each combination results in different fraction of case and control specific nodes. These results can be compared to study the effect of absence of each SNP. The fraction of the genotype from each set is then calculated and the comparative results are plotted in Figure 5. If after removal of a particular SNP (SNP4) from ACS-control population, the number of case specific genotypes observed from the main population set decreases compared to the others and removal of SNP3 on the other hand, decreases the control specific genotypes, then SNP 4 and SNP3 might be considered as a probable risk SNP and protective SNP respectively. This phenomenon has been also observed in case of oral cancer and control samples. Here, removal of SNP2 and SNP4 decrease the case specific and control specific population fraction respectively (Figure S1 in File S1). We have checked the statistical significance of the numbers we obtained as frequencies from both the ACS and oral cancer. It is found that when we calculate the chi-square test with one SNP omission (in ACS) the p values are not significant but omission of SNP4 (reduces case specific population) and SNP3 (reduces control specific population) give the p values which are very much closer to the significance level. Therefore it can be said that if we increase the sample size the difference might be statistically significant which is supported by the fact that in case of oral cancer (where sample size was higher) removal of SNP2 significantly decreases the case specific genotypic fraction (p=0.034) and removal of SNP4 significantly decreases the control specific genotypic fraction (p=0.032) (Table S3 in File S1). Therefore SNP2 seems to be the ‘risk SNP’ and SNP4 seems to be the ‘protective SNP’ for oral cancer (OR values are not given here, since OR > 1 is risk, OR< 1 is protective). Different networks have been generated depending upon the removal of one locus at a time from the whole population and the width of the edges represents the change of the number of individuals in specific cluster (Figure S2 in File S1). It would be identified more clearly when we compare these networks with the network that has been generated with all 5 loci (Figure 6). The frequency of genotypic combinations obtained after one locus omission in ACS-control and in oral cancer-leukoplakia-control is represented through Tables S4.A and S4.B in File S1, respectively.

Similarly, we have also studied how fraction of ACS-case and control specific population varies with different genotypic combinations as mentioned above after removal of one locus. The effects in the population fraction after omission of one locus are observed in Figure S3 in File S1. Here also, SNP4 (i.e. *P2RY12* 1622C>*T*) is maintaining its importance since the fraction of case specific population decrease after removal of that SNP.

#### Formulation of an optimization principle and Identification of important SNP

It is evident from Figure 5 that when SNP4 (*P2RY12*, 1622C>*T*) is removed, the fraction of cases (ACS) decreases to almost 20% of the total population (i.e., case and control), while the same value remains ~ 40% for the rest of the sets (i.e. the case fraction is 40%) in the main population set.

This method has also been validated in the oral cancer data and we have found that there are some particular SNPs whose presence or absence can change the fraction of specific genotype remarkably (Figure S1 in File S1). Although, statistically, no significant association between disease (cancer, precancer) and polymorphism was observed (data not shown) [16] but Figure S1 in File S1 shows that there are some definite SNPs which can control number of diseased and healthy individuals in the total population, for example removal of SNP2 (*XRCC1, codon 399, Arg/Gln*) decreases the case specific genotypic fraction and removal of SNP4 (*XPC, Codon 939, Lys/Gln*) decreases the control specific genotypic fraction which corroborates partially our previous report that polymorphism at *XRCC1* increases risk of cancer [16]. Therefore, this simple network can also propose risk or protective genotype in a case-control population.

#### Design of a network based on combination of genotypes

While the above data did the risk prediction of a genotype at a single polymorphic position, the network analysis revealed a greater inclination of certain genotypic sets towards case population (for e.g., genotypic set 22 22 12 12 11) or control population (for e.g., genotypic set 22 22 12 11 11). The risk of the disease is evidently high in certain genotypic sets. This is seen by the relative edge distribution of the original case control network as seen in Figure 6.

In this network, combination of the 5 SNPs in each individual results in 35 unique genotype sets (among 35 =243 possible sets) which may act as potential nodes. The exclusion of other possible nodes may depend on (i) actual exclusion of certain genotypic combination from the population due to selection process (ii) incompleteness of the study set. However, the second possibility can be eliminated by increase of sample size. The nodes are then connected to two major nodes namely case and control. This simple description helps in the following ways.

#### Visual representation of relationship between combined genotypes

**Figure 7:**
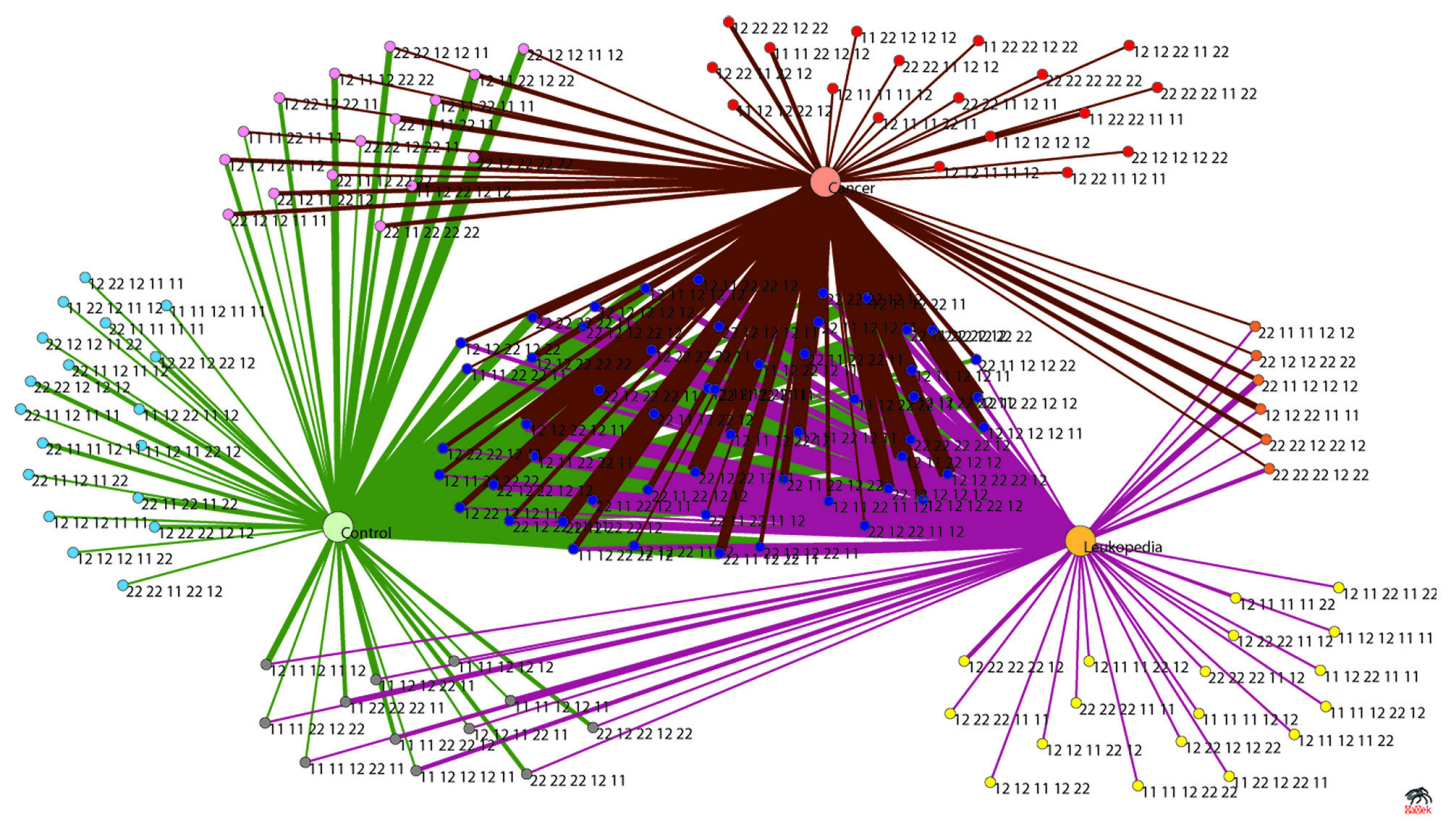
Network representation of segregation of combined genotypic data of three population (oral cancer, leukoplakia and control). Five SNPs (studied with leukoplakia, cancer and control samples) of each individual are combined to form super-set genotypes. One hundred fourty three unique genotype-sets are observed of which 18 are specific to each control, leukoplakia and cancer individuals, 12 genotype-sets are present in both control and leukoplakia individuals, 18 genotype-sets are present in both control and cancer individuals, only 6 genotype-sets are common between leukoplakia and cancer individuals and as many as 53 genotype-sets are common to case, control and leukoplakia. The number of occurrences of each particular genotype-set is illustrated through its corresponding edge-width. The circles in i) red ii) violet iii) yellow iv) prussian blue v) grey vi) orange and vii) blue respectively represents the following groups i) cancer only ii) cancer and control iii) leukoplakia only iv) control only v) control and leukoplakia vi) cancer and leukoplakia and vii) cancer, leukoplakia and control.

Out of the 35 observed genotype sets 14 are specific to ACS cases (marked as red nodes in the graphs), 7 are specific to ACS controls (marked as green nodes) and 14 are present in cases as well as controls (marked as brown nodes). However, the number of occurrences of these 14 common genotypes varies in case and controls (see the edge- width in Figure 6). Figure 6 allows one to track genotypes more susceptible to disease. Owing to the smaller sample size in our ACS case-control study, we have performed a similar network study on another case-control data set which involves 369 controls, 219 leukoplakia (pre cancer) and 298 oral cancer cases and a clear segregation of 3 sub-population (main population is divided into three groups) with their specific genotypes (with a ‘common’ fraction between all the sub-populations) is occurring in the network (Figure 7).

**Table 3:** Position wise rank conservation of Case-control Ratio in random data sets where Type 1 datasets are generated using simulation method which conserves genotypic frequencies while Type 2 datasets have conserved allele frequencies.

**Case Control Ratio- Rank in original dataset (SNP Number)**	**SNP wise Rank conservation (%)**	
	**Type 1 Dataset**	**Type 2 Dataset**
1st (SNP4)	87.6	99.2
2nd (SNP1)	25.6	43
3rd (SNP5)	21.2	25.8
4th (SNP2)	30.8	27
5th (SNP3)	12.4	20

#### Ranking of SNPs based on the simulation

The idea behind the two methods of simulation with a given dataset of ACS-control is that in either case, we have not altered the allele frequencies of the individual SNPs in random datasets; consequently the chi-square value has also not been changed. So, we can directly compare the existing method of risk SNP prediction with our method. From Tables 3 and 4, we can suggest the following:

1 The fact that these data sets have same chi-square value, but changing ranks in the random datasets, suggest that this method might be somehow more sensitive than the conventional statistical analysis.
2 The important risk SNPs predicted by this method is robust enough since it is reflected in all the shuffled genotypes superset datasets. This means that the method is affected by individual allele frequency as well as it shows the importance of combinatorial effect of different SNPs.

**Figure 8:**
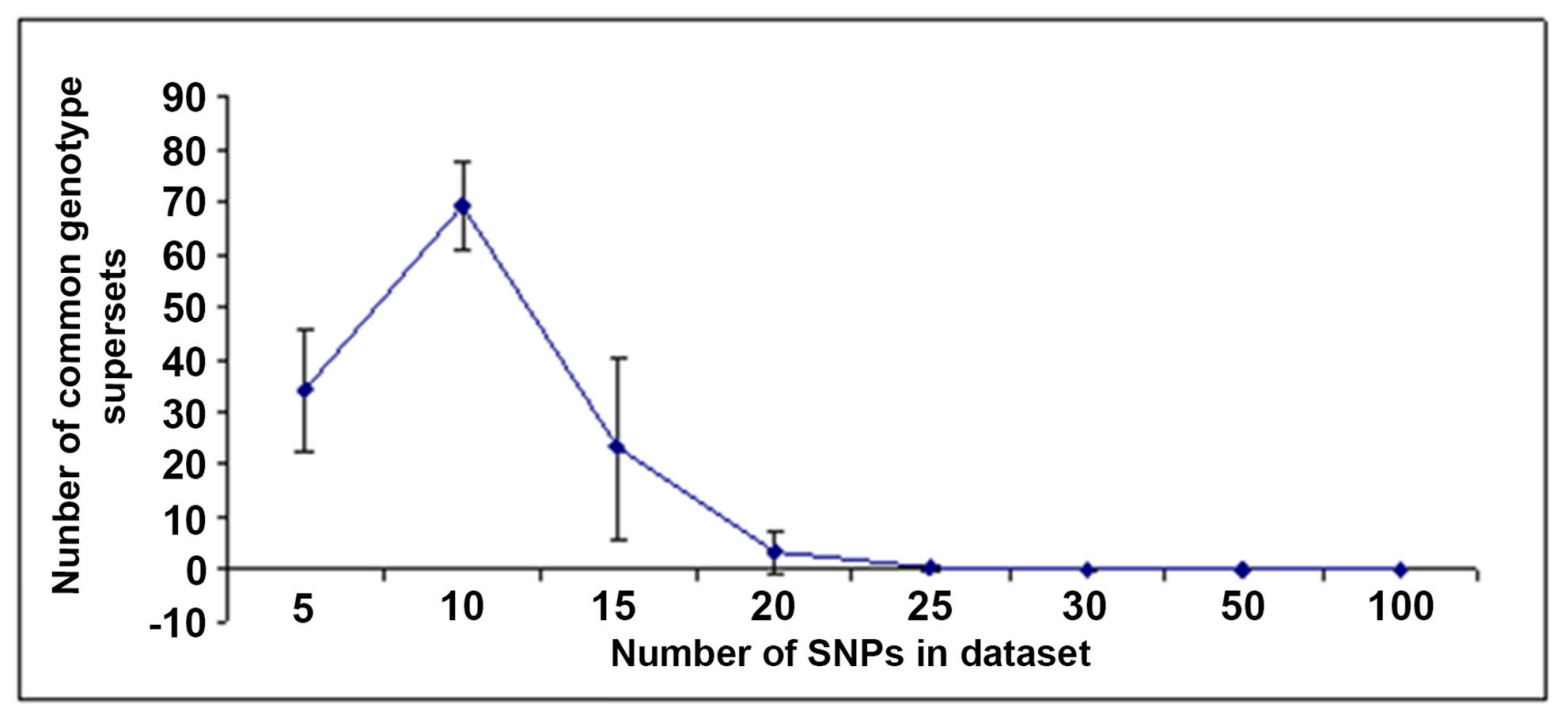
Number of common genotypes supersets plotted as a function of SNPs. A simulation study has been performed with 5, 10, 15, 20, 25, 30, 50, 100, 150, 200, 250 SNPs (SNP subset). They are picked up randomly from a dataset of 1804 SNPs of 635 individuals. The error bar shows the variations resulting from 1000 simulations.

The number of common genotype supersets in case of large dataset is plotted as a function of number of SNPs (Figure 8). The figure clearly shows that the number of common genotypes increases till 10 SNPs, followed by a sharp decay as the number of SNPs is increased and after reaching 25 SNPs, the number of case specific and control specific populations totally segregates and there are no more common genotypes between cases and controls. In other words, in such cases individuals become uniquely marked. Therefore it can be said that for a particular sample size of the population there might be a definite number of SNPs till which we can segregate the population in case, control and common groups.

**Table 4:** Position wise rank conservation of case specific nodes in random data sets where Type 1 datasets are generated using simulation method which conserves genotypic frequencies while Type 2 datasets have conserved allele frequencies.

**Case specific node- Rank in original dataset (SNP Number)**	**Position wise Rank conservation (%)**	
	**Type 1 Dataset**	**Type 2 Dataset**
1st (SNP4)	100	100
2nd (SNP1)	26.2	46
3rd (SNP5)	18.6	30
4th (SNP2)	36.6	29.6
5th (SNP3)	9.6	19.8

### Comparative Appraisal

Interestingly it has been found that from network approach the most important SNP whose absence affects the case–control ratio in the ACS case-control study is SNP 4 (*P2RY12* 1622C>T) and simulation of the whole genotypic data also shows that SNP4 ranks first when we make position wise SNP conservation ranking. Conventional statistical analysis shows that SNP4 is important polymorphism which is significantly associated with disease. Similarly these methods, however, are partially consistent with the results obtained after statistical analysis ([16] and data not shown) in case of the oral cancer studies (Figure S1 in File S1).

We have found that the LD values of SNPs related to ACS, estimated as R^2^, are not strong (except LD between SNP3 and SNP4) and in case of oral cancer the SNPs are not present on the same chromosome and there was no LD among them [16]. So, there is possibility that certain combination/s of genotype/s at SNP3 and SNP4 might be more frequent in ACS cases and controls. It has been observed that all combinations of 5 genotypes (at SNP1, SNP2, SNP3, SNP4 and SNP5) in which 12 12 genotypes are present at SNP3 and SNP4 positions, respectively, are overrepresented in ACS patients whereas all combinations of 5 genotypes (at SNP1, SNP2, SNP3, SNP4 and SNP5) in which 12 11 genotypes are present at SNP3 and SNP4 positions, respectively, are overrepresented in ACS control. These results corroborate with the results of haplotype data (Table 2) which shows that 2-2 haplotype is more frequent in ACS patients and 2-1 haplotype is more frequent in ACS controls (data not shown). In this study, we have proposed an approach of combined genotypic analysis to signify the independent distribution of genotypes instead of doing haplotypic model. If there is strong LD among SNPs, a haplotypic model can be generated where the linked SNPs would co-appear. While the proposed methodology is independent of the LD values, knockout method provides some information regarding SNP co-occurrence patterns.

## Discussion

Perturbations of platelet’s function may lead to pathological thrombus formation and vascular occlusion, resulting in heart stroke, myocardial infarction or acute coronary syndrome (ACS) [4]. After disruption of atherosclerotic plaques, platelet aggregation also plays an important role in development of myocardial infarction and other acute coronary syndromes [4]. Binding of platelets to von Willebrand factor and collagen is the initiating event in platelet activation and it leads to platelet degranulation and release of platelet agonists (ADP, ATP, Serotonin) [22].

Platelets contain at least five purinergic G protein-coupled receptors, e.g., the pro-aggregatory P2Y(1) and P2Y(12) receptors, a P2Y(14) receptor of unknown function, and anti-aggregatory A(2A) and A(2B) adenosine receptor (ARs), in addition to the ligand-gated P2X1 ion channel [23]. P2RY1 initiates platelet shape change [24] and micro aggregation formation through the mobilization of internal calcium stores. P2RY12 is coupled to adenylyl cyclase inhibition [25] and is essential for a full aggregation response to ADP and the stabilization of aggregates has been shown to initiate platelet activation when stimulated in concert. Variability in platelet response to anti-platelet drugs and their clinical relevance have been well described [26] in literature, although the underlying mechanism remains unclear. It was observed later that there are remarkable inter-individual differences in the response of diseased individuals toward anti-platelet drug therapy and the difference is reproducible over time [27]. Again the propensity of higher platelet aggregation even in control population is an attribute that deserves attention.

The present study primarily addressed the question of association between ACS disease and presence of important SNPs or genotypes in patients (using both network and conventional statistical approach). Our results showed that *A* allele at 1622 A>*G* (*P2RY1*) and *T* allele at 1622 C>*T* (P2RY12) could be risk factors for ACS disease. Again, *T-T* haplotype (Table 2), which includes, *T* allele at 1622 C>*T* (P2RY12), also increased the risk of disease. The additional question concerning association between aggregability of platelets and SNPs has not been addressed. However, our preliminary result on aggregability of platelets in controls with small sample size (n=26) indicates that platelet aggregation is more in individuals with *AA* genotypes at *P2RY1* (*1622 A>G*), than that in individuals with *GG* genotype. There is a decreasing trend, though not statistically significant, in aggregation of platelets in controls with *AA*>*AG*>*GG* genotypes (65+ 30, 51+42, 44+20 respectively). This result of platelet aggregation in controls also corroborates the result in patients (Table 2) which shows that *A*-allele is more frequent in patients than that in controls (*A*=0.23 and 0.15, respectively). So, individuals with *AA* genotype may be more prone to ACS compare to individuals with *GG* genotype.

As ACS is becoming one of the leading causes of mortality in the Indian population, in this study we have tried to find out some risk factors behind the occurrence of the disease in Eastern Indian population for the first time. It is known that platelet aggregation in the blood vessel causes myocardial infarction and after anti-platelet-aggregation drug therapy at the initial level usually patients get rid off from the risk of heart block. But the aggregation alone may not successfully predict the risk.

We have been able to segregate the genotypes of diseased and healthy individuals using the proposed network containing super-nodes (disease stages) and nodes (genotypes). We have also shown the effectiveness of a simple optimization algorithm in which restructuring the nodes due to missing genotype alters the case-control distribution and the particular genotype whose absence causes maximal shift to case or control distribution (Figure 5) would be considered as the one corresponding to maximal and minimal risk of the disease.

The Network based approach is able to mine the important one among the 5 SNPs in oral cancer, a feat that was not fully achievable by conventional statistical methods ([16] and unpublished data). It shows that some genotype combinations are common in control, precancer and cancer individuals, so there is possibility that these common genotypes are susceptible to precancer and/or cancer provided individuals are exposed to tobacco carcinogens (Figure 7 and Table S3 in File S1). Some genotype combinations are specific to control, precancer and cancer individuals. These combinations of genotypes in controls may be resistant to precancer or cancer even after tobacco exposure as we know some individuals do not progress to disease even after tobacco exposures for long time with sufficient tobacco dose. Few genotype combinations which are specific to leukoplakia, may not lead to cancer since only ~5-10% of leukoplakia patients may progress to cancer. Again, few combinations of genotypes which are specific for cancer patients may be very susceptible to cancer after exposure to tobacco carcinogens and these genotype combinations are not frequent in the population as these combinations are not present in control as well as leukoplakia patients (Figure 7). So, network based presentation of genotypes and stage of disease gave clear picture of distribution of genotypes among patients and controls. Number of cancer individuals reduced when *XRCC1* (i.e. SNP2) is omitted from genotype combinations (Figure S1 in File S1) and it indicates that presence *XRCC1* polymorphism may increase risk of cancer among population. This result is also corroborating our previous report that *XRCC1* polymorphism increased the risk of oral cancer [16]. The correctness of the Network based approach is validated in case of the ACS based patient data in which the Statistical prediction matches exactly with the Network based prediction. SNP number specificity which has added a great impact in our network based case-control study helps in segregating unique case and control specific individuals after a certain SNP number (Figure 8). The representation of genotypic data through network is able to show segregated data clusters on protective and risk genotypes as well as mixed genotypes. In future perspective, this type of network architecture can also be used in other datasets to predict various dimensions. While the observation regarding ACS and the cancer and precancer states are new, the methodology developed can also be integrated into a supervised learning algorithm, in which with a given genotype of a clinically uncharacterized individual it would be possible to predict the risk.

For diseases with large SNPs the method needs a screening module (not described yet) in which significant SNPs are gradually sorted out in increments. The screened SNPS then can be subjected to further iterative screening using a similar network based method. As the method is modular in nature the proposed extensions seems feasible.

## Supporting Information

File S1 Supporting figures and tables. **Figure S1, One polymorphic site is removed at a time from all the genotypic sets in the population to predict the probable risk allele.** The effect is studied in terms of the number of unique cases with precancer (leukoplakia) and cancer remaining after each locus deletion. The number under each bar in the X axis represents the omitted locus. A) Exclusion of SNP2 shows highest decline in the size of population suffering from cancer. B) Deletion of any locus is not associated with increase or decrease in precancerous population. C) Exclusion of SNP4 shows highest decline in the size of healthy (control) population. Figure S2, Restructuring of the case-control specific genotypes as different supersets are created taking 4 SNPs at a given time, the one removed each time is denoted by "*". Figure S3, One SNP at a time is removed from all the genotypic set in the ACS-control population to observe the effect after omission of one locus. The removed locus is denoted by * in the genotype supersets taking 4 loci at a time. The effect is studied in terms of the distribution of population under different conditions namely Case, Control and Common groups. **Table S1, Polymorphisms identified in P2RY1 and P2RY12 genes. Table S2**, A. Frequency of combination of genotypes among ACS patients, respective controls and combined individuals. B. Frequency of combination of genotypes among oral cancer patients, precancer patients and controls. **Table S3, p values after omission of one SNP from oral cancer and control population.** Omission of SNP2 and SNP4 significantly decrease the case specific and control specific genotypic fraction respectively. Therefore SNP2 might be called as ‘risk SNP’ and SNP4 as ‘protective SNP’. The significant p-values are marked as ‘*’. **Table S4**, A. Frequency of combination of genotypes obtained after omission of one SNP from ACS and control population B. Frequency of combination of genotypes obtained after omission of one SNP from oral cancer, leukoplakia and control population. Omitted SNPs are marked as ‘*’.
(DOC)
