# Supplementary material for: Single Nucleotide Polymorphism Network: A Combinatorial Paradigm for Risk Prediction
Source: PLoS One. 2013 Sep 11;8(9):e74067. doi: 10.1371/journal.pone.0074067 (PMC3770707; doi:10.1371/journal.pone.0074067)
Supplement: File S1 — Figure S1, One polymorphic site is removed at a time from all the genotypic sets in the population to predict the probable risk allele. The effect is studied in terms of the number of unique cases with precancer (leukoplakia) and cancer remaining after each locus deletion. The number under each bar in the X axis represents the omitted locus. A) Exclusion of SNP2 shows highest decline in the size of population suffering from cancer. B) Deletion of any locus is not associated with increase or decrease in precancerous population. C) Exclusion of SNP4 shows highest decline in the size of healthy (control) population. Figure S2, Restructuring of the case-control specific genotypes as different supersets are created taking 4 SNPs at a given time, the one removed each time is denoted by "*". Figure S3, One SNP at a time is removed from all the genotypic set in the ACS-control population to observe the effect after omission of one locus. The removed locus is denoted by * in the genotype supersets taking 4 loci at a time. The effect is studied in terms of the distribution of population under different conditions namely Case, Control and Common groups. Table S1, Polymorphisms identified in P2RY1 and P2RY12 genes. Table S2, A. Frequency of combination of genotypes among ACS patients, respective controls and combined individuals. B. Frequency of combination of genotypes among oral cancer patients, precancer patients and controls. Table S3, p values after omission of one SNP from oral cancer and control population. Omission of SNP2 and SNP4 significantly decrease the case specific and control specific genotypic fraction respectively. Therefore SNP2 might be called as ‘risk SNP’ and SNP4 as ‘protective SNP’. The significant p-values are marked as ‘*’. Table S4, A. Frequency of combination of genotypes obtained after omission of one SNP from ACS and control population B. Frequency of combination of genotypes obtained after omission of one SNP from oral cancer, leukoplakia and cont [file pone.0074067.s001.doc]

**Figure S1** **represents the cancer, precancer and control specific genotypic fraction in case of every single locus omission**

**Figure S1:** One polymorphic site is removed at a time from all the genotypic sets in the population to predict the probable risk allele. The effect is studied in terms of the number of unique cases with precancer (leukoplakia) and cancer remaining after each locus deletion. The number under each bar in the X axis represents the omitted locus.

A) Exclusion of **SNP2** shows highest decline in the size of population suffering from cancer.

B) Deletion of any locus is not associated with increase or decrease in precancerous population

C) Exclusion of **SNP4** shows highest decline in the size of healthy (control) population.

**Figure S2 shows the network representation of the genotypic combination after omission of one locus**

**
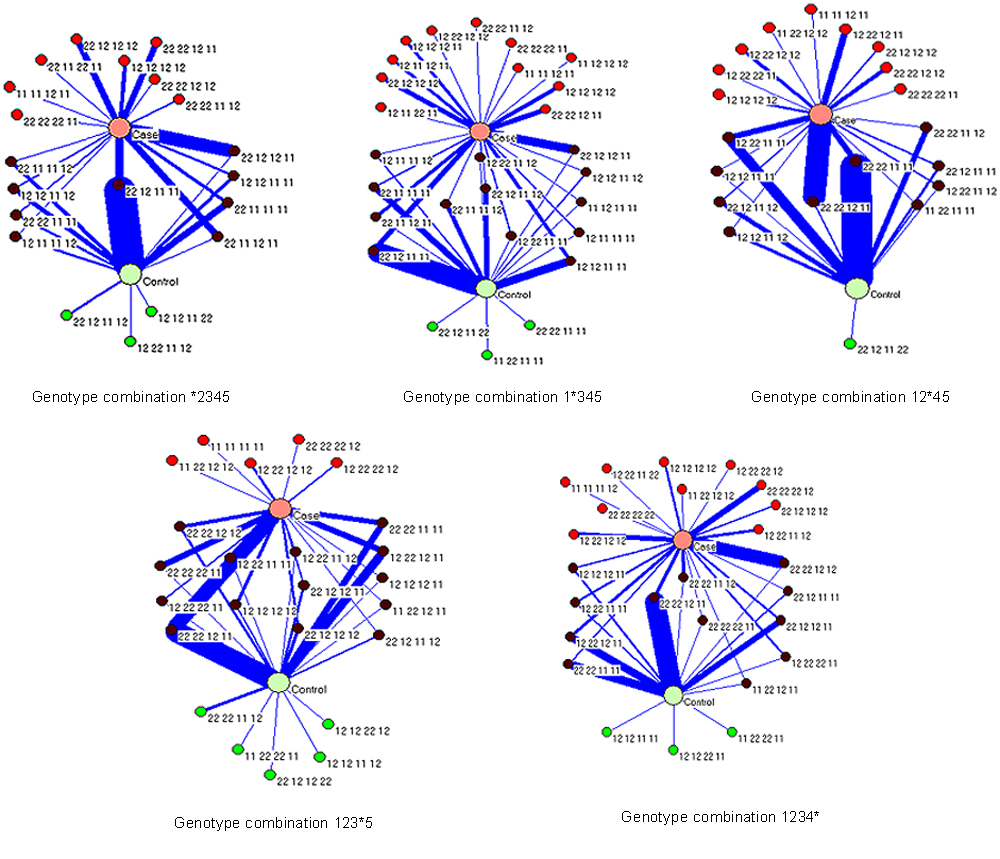
**

**Figure S2.** Restructuring of the case-control specific genotypes as different supersets are created taking 4 SNPs at a given time, the one removed each time is denoted by '’*".

**Figure S3 represents the fraction of population after removal of one locus at a time**

**Figure S3.**  One SNP at a time is removed from all the genotypic set in the ACS-control population to observe the effect after omission of one locus. The removed locus is denoted by * in the genotype supersets taking 4 loci at a time. The effect is studied in terms of the distribution of population under different conditions namely Case, Control and Common groups.

**Table S1. Polymorphisms identified in *P2RY1* and *P2RY12* genes**

| **Gene** | **Location** | **Nucleotide position** | **NCBI dbSNP reference** | **Obsereved alleles in ACS (Approximate frequencies)** | **Obsereved alleles in control (Approximate frequencies)** |
| --- | --- | --- | --- | --- | --- |
| *P2RY1* | Coding | 1622 | rs701205 | A/G  0.23/0.77 | A/G  0.15/0.85 |
| *P2RY12* | Coding | 234 | rs6785930 | T/C  0.53/0.47 | T/C  0.58/0.42 |
|  | Intron B | 742 | rs2046934 | T/C  0.11/0.89 | T/C  0.18/0.82 |
|  | Downstream | 1622 | - | C/T  0.60/0.40 | C/T  0.97/0.03 |
|  | Downstream | 2014 | rs6803224 | C/T  0.84/0.16 | C/T  0.86/0.14 |

**Table S2.A. Frequency of combination of genotypes among ACS patients, respective controls and combined** individuals

| **Case** | |  | **Control** | |  | **Combined** | |
| --- | --- | --- | --- | --- | --- | --- | --- |
| SNP combination | Freq |  | SNP combination | Freq |  | SNP combination | Freq |
| 22 22 12 12 11 | 13 |  | 22 22 12 11 11 | 26 |  | 11 11 11 12 11 | 1 |
| 22 22 22 12 11 | 8 |  | 12 22 12 11 11 | 11 |  | 11 22 12 11 11 | 2 |
| 22 22 11 12 11 | 5 |  | 22 22 11 11 11 | 8 |  | 11 22 12 12 12 | 1 |
| 22 22 12 11 11 | 5 |  | 22 12 12 11 12 | 4 |  | 11 22 22 11 11 | 1 |
| 22 22 12 12 12 | 5 |  | 22 22 11 11 12 | 4 |  | 12 12 11 11 12 | 1 |
| 12 22 11 12 11 | 4 |  | 22 12 12 11 11 | 3 |  | 12 12 12 11 11 | 2 |
| 12 22 12 11 11 | 4 |  | 22 22 12 11 12 | 3 |  | 12 12 12 11 12 | 3 |
| 12 22 12 12 11 | 4 |  | 12 12 12 11 12 | 2 |  | 12 12 12 12 12 | 3 |
| 12 12 12 12 12 | 3 |  | 22 12 11 11 12 | 2 |  | 12 12 22 11 12 | 1 |
| 12 22 12 12 12 | 3 |  | 11 22 12 11 11 | 1 |  | 12 22 11 11 11 | 3 |
| 12 22 11 11 11 | 2 |  | 11 22 22 11 11 | 1 |  | 12 22 11 11 12 | 2 |
| 12 22 22 11 11 | 2 |  | 12 12 11 11 12 | 1 |  | 12 22 11 12 11 | 4 |
| 22 12 12 11 11 | 2 |  | 12 12 12 11 11 | 1 |  | 12 22 11 22 11 | 1 |
| 22 12 12 12 12 | 2 |  | 12 12 22 11 12 | 1 |  | 12 22 12 11 11 | 15 |
| 22 22 11 11 11 | 2 |  | 12 22 11 11 11 | 1 |  | 12 22 12 12 11 | 4 |
| 11 11 11 12 11 | 1 |  | 12 22 11 11 12 | 1 |  | 12 22 12 12 12 | 3 |
| 11 22 12 11 11 | 1 |  | 12 22 22 11 11 | 1 |  | 12 22 22 11 11 | 3 |
| 11 22 12 12 12 | 1 |  | 22 12 12 11 22 | 1 |  | 12 22 22 11 12 | 1 |
| 12 12 12 11 11 | 1 |  | 22 22 11 12 11 | 1 |  | 12 22 22 12 12 | 1 |
| 12 12 12 11 12 | 1 |  | 22 22 12 12 11 | 1 |  | 22 12 11 11 12 | 3 |
| 12 22 11 11 12 | 1 |  | 22 22 22 11 11 | 1 |  | 22 12 12 11 11 | 5 |
| 12 22 11 22 11 | 1 |  |  |  |  | 22 12 12 11 12 | 5 |
| 12 22 22 11 12 | 1 |  |  |  |  | 22 12 12 11 22 | 1 |
| 12 22 22 12 12 | 1 |  |  |  |  | 22 12 12 12 12 | 2 |
| 22 12 11 11 12 | 1 |  |  |  |  | 22 22 11 11 11 | 10 |
| 22 12 12 11 12 | 1 |  |  |  |  | 22 22 11 11 12 | 4 |
| 22 22 22 11 12 | 1 |  |  |  |  | 22 22 11 12 11 | 6 |
| 22 22 22 22 11 | 1 |  |  |  |  | 22 22 12 11 11 | 31 |
|  |  |  |  |  |  | 22 22 12 11 12 | 3 |
|  |  |  |  |  |  | 22 22 12 12 11 | 14 |
|  |  |  |  |  |  | 22 22 12 12 12 | 5 |
|  |  |  |  |  |  | 22 22 22 11 11 | 1 |
|  |  |  |  |  |  | 22 22 22 11 12 | 1 |
|  |  |  |  |  |  | 22 22 22 12 11 | 8 |
|  |  |  |  |  |  | 22 22 22 22 11 | 1 |

**Table S2.B. Frequency of combination of genotypes among oral cancer patients, precancer patients and controls**

**Control Cancer Leukoplakia**

| **SNP combination** | **Frequency** |  | **SNP combination** | **Frequency** |  | **SNP combination** | **Frequency** |
| --- | --- | --- | --- | --- | --- | --- | --- |
| 1111121111 | 1 |  | 1111221111 | 1 |  | 1111111212 | 1 |
| 1111121211 | 1 |  | 1111221212 | 2 |  | 1111121211 | 2 |
| 1111121212 | 2 |  | 1111222211 | 1 |  | 1111121212 | 1 |
| 1111122211 | 1 |  | 1112121212 | 1 |  | 1111122211 | 1 |
| 1111221111 | 1 |  | 1112122212 | 2 |  | 1111122212 | 1 |
| 1111221222 | 1 |  | 1112221211 | 4 |  | 1111122222 | 1 |
| 1111222211 | 4 |  | 1112221212 | 3 |  | 1111221222 | 1 |
| 1111222212 | 2 |  | 1112222211 | 1 |  | 1111222211 | 2 |
| 1112112212 | 1 |  | 1112222212 | 3 |  | 1111222212 | 1 |
| 1112121211 | 1 |  | 1122121212 | 1 |  | 1112121111 | 1 |
| 1112122211 | 2 |  | 1122221111 | 2 |  | 1112121211 | 2 |
| 1112221112 | 1 |  | 1122221222 | 1 |  | 1112122211 | 1 |
| 1112221211 | 3 |  | 1211111112 | 1 |  | 1112221111 | 1 |
| 1112221212 | 3 |  | 1211112211 | 1 |  | 1112221211 | 1 |
| 1112222211 | 1 |  | 1211121211 | 6 |  | 1112222211 | 1 |
| 1112222212 | 1 |  | 1211121212 | 3 |  | 1112222212 | 2 |
| 1122121112 | 1 |  | 1211122211 | 7 |  | 1122122211 | 1 |
| 1122222211 | 2 |  | 1211122212 | 6 |  | 1122222211 | 2 |
| 1211121112 | 3 |  | 1211122222 | 1 |  | 1211111122 | 1 |
| 1211121211 | 2 |  | 1211221111 | 2 |  | 1211112212 | 1 |
| 1211121212 | 8 |  | 1211221112 | 2 |  | 1211121112 | 1 |
| 1211122211 | 6 |  | 1211221211 | 7 |  | 1211121122 | 1 |
| 1211122212 | 4 |  | 1211221212 | 8 |  | 1211121211 | 3 |
| 1211122222 | 3 |  | 1211221222 | 2 |  | 1211121212 | 3 |
| 1211221111 | 3 |  | 1211222211 | 8 |  | 1211122211 | 3 |
| 1211221112 | 1 |  | 1211222212 | 7 |  | 1211122212 | 3 |
| 1211221211 | 11 |  | 1211222222 | 2 |  | 1211221112 | 1 |
| 1211221212 | 7 |  | 1212111112 | 1 |  | 1211221122 | 1 |
| 1211221222 | 5 |  | 1212121112 | 2 |  | 1211221211 | 6 |
| 1211222211 | 11 |  | 1212121211 | 5 |  | 1211221212 | 4 |
| 1211222212 | 5 |  | 1212121212 | 2 |  | 1211222211 | 6 |
| 1211222222 | 1 |  | 1212122211 | 5 |  | 1211222212 | 9 |
| 1212112211 | 1 |  | 1212122212 | 4 |  | 1211222222 | 1 |
| 1212121111 | 1 |  | 1212122222 | 1 |  | 1212111222 | 1 |
| 1212121112 | 2 |  | 1212221111 | 3 |  | 1212112211 | 1 |
| 1212121122 | 1 |  | 1212221112 | 1 |  | 1212112212 | 1 |
| 1212121211 | 6 |  | 1212221122 | 1 |  | 1212121211 | 5 |
| 1212121212 | 1 |  | 1212221211 | 10 |  | 1212121212 | 3 |
| 1212122211 | 4 |  | 1212221212 | 7 |  | 1212122211 | 3 |
| 1212122212 | 3 |  | 1212221222 | 2 |  | 1212122212 | 3 |
| 1212122222 | 2 |  | 1212222211 | 8 |  | 1212122222 | 1 |
| 1212221112 | 4 |  | 1212222212 | 8 |  | 1212221111 | 1 |
| 1212221211 | 12 |  | 1212222222 | 3 |  | 1212221112 | 1 |
| 1212221212 | 8 |  | 1222111211 | 1 |  | 1212221211 | 7 |
| 1212221222 | 3 |  | 1222112212 | 1 |  | 1212221212 | 1 |
| 1212222211 | 12 |  | 1222121211 | 3 |  | 1212221222 | 2 |
| 1212222212 | 6 |  | 1222122211 | 1 |  | 1212222211 | 6 |
| 1212222222 | 2 |  | 1222221211 | 2 |  | 1212222212 | 3 |
| 1222121111 | 1 |  | 1222221222 | 2 |  | 1212222222 | 1 |
| 1222121211 | 3 |  | 1222222211 | 1 |  | 1222121211 | 1 |
| 1222122211 | 2 |  | 2211111212 | 1 |  | 1222121222 | 1 |
| 1222122212 | 2 |  | 2211112211 | 2 |  | 1222221111 | 1 |
| 1222221211 | 2 |  | 2211121211 | 7 |  | 1222221112 | 1 |
| 1222221212 | 2 |  | 2211121212 | 2 |  | 1222221211 | 1 |
| 1222222211 | 3 |  | 2211121222 | 2 |  | 1222222211 | 2 |
| 2211111111 | 1 |  | 2211122211 | 5 |  | 1222222212 | 2 |
| 2211111211 | 2 |  | 2211122212 | 5 |  | 2211111212 | 1 |
| 2211112211 | 1 |  | 2211122222 | 1 |  | 2211121211 | 2 |
| 2211121111 | 1 |  | 2211221111 | 1 |  | 2211121212 | 3 |
| 2211121112 | 2 |  | 2211221112 | 1 |  | 2211121222 | 1 |
| 2211121122 | 1 |  | 2211221211 | 8 |  | 2211122211 | 5 |
| 2211121211 | 6 |  | 2211221212 | 3 |  | 2211122212 | 4 |
| 2211121222 | 2 |  | 2211221222 | 4 |  | 2211221111 | 1 |
| 2211122211 | 6 |  | 2211222211 | 11 |  | 2211221112 | 1 |
| 2211122212 | 3 |  | 2211222212 | 7 |  | 2211221211 | 7 |
| 2211122222 | 2 |  | 2211222222 | 2 |  | 2211221212 | 8 |
| 2211221111 | 2 |  | 2212112212 | 2 |  | 2211221222 | 1 |
| 2211221112 | 1 |  | 2212121111 | 2 |  | 2211222211 | 8 |
| 2211221122 | 2 |  | 2212121112 | 1 |  | 2211222212 | 2 |
| 2211221211 | 13 |  | 2212121211 | 5 |  | 2212121211 | 2 |
| 2211221212 | 4 |  | 2212121212 | 6 |  | 2212121212 | 1 |
| 2211221222 | 1 |  | 2212121222 | 1 |  | 2212122211 | 1 |
| 2211222211 | 17 |  | 2212122211 | 2 |  | 2212122212 | 7 |
| 2211222212 | 11 |  | 2212122212 | 2 |  | 2212122222 | 1 |
| 2211222222 | 2 |  | 2212122222 | 1 |  | 2212221111 | 4 |
| 2212112212 | 1 |  | 2212221111 | 2 |  | 2212221112 | 1 |
| 2212121111 | 1 |  | 2212221112 | 1 |  | 2212221211 | 9 |
| 2212121112 | 4 |  | 2212221211 | 8 |  | 2212221212 | 5 |
| 2212121122 | 1 |  | 2212221212 | 8 |  | 2212221222 | 1 |
| 2212121211 | 3 |  | 2212222211 | 10 |  | 2212222211 | 8 |
| 2212121212 | 5 |  | 2212222212 | 6 |  | 2212222212 | 3 |
| 2212122211 | 9 |  | 2212222222 | 5 |  | 2222122212 | 1 |
| 2212122212 | 10 |  | 2222111211 | 1 |  | 2222221111 | 1 |
| 2212221111 | 1 |  | 2222111212 | 1 |  | 2222221112 | 1 |
| 2212221112 | 2 |  | 2222121211 | 1 |  | 2222221211 | 1 |
| 2212221211 | 9 |  | 2222122211 | 1 |  | 2222221212 | 2 |
| 2212221212 | 17 |  | 2222122212 | 2 |  | 2222221222 | 2 |
| 2212221222 | 2 |  | 2222221122 | 1 |  | 2222222211 | 4 |
| 2212222211 | 15 |  | 2222221212 | 1 |  | 2222222212 | 4 |
| 2212222212 | 7 |  | 2222221222 | 1 |  | Grand Total | 219 |
| 2212222222 | 3 |  | 2222222211 | 3 |  |  |  |
| 2222112212 | 1 |  | 2222222212 | 2 |  |  |  |
| 2222121211 | 3 |  | 2222222222 | 1 |  |  |  |
| 2222121212 | 2 |  | Grand Total | 298 |  |  |  |
| 2222122211 | 1 |  |  |  |  |  |  |
| 2222221211 | 2 |  |  |  |  |  |  |
| 2222221212 | 2 |  |  |  |  |  |  |
| 2222222211 | 5 |  |  |  |  |  |  |
| 2222222212 | 1 |  |  |  |  |  |  |
| Grand Total | 369 |  |  |  |  |  |  |
|  |  |  |  |  |  |  |  |
|  |  |  |  |  |  |  |  |

**Table S3. p values after omission of one SNP from oral cancer and control population**

| **Omission of one SNP** | **p values for case specific genotypic fraction** | **p values for control specific genotypic fraction** |
| --- | --- | --- |
| **1** | **0.9** | **0.451** |
| **2** | **0.034*** | **0.517** |
| **3** | **0.223** | **0.223** |
| **4** | **0.7** | **0.032*** |
| **5** | **0.6** | **0.618** |

**Omission of SNP2 and SNP4 significantly decrease the case specific and control specific genotypic fraction respectively. Therefore SNP2 might be called as ‘risk SNP’ and SNP4 as ‘protective SNP’. The significant p-values are marked as ‘*’.**

**Table S4.A. Frequency of combination of** genotypes obtained after omission of one SNP from ACS and control population

| **SNP combination** | **Case specific**  **genotypic fraction** | **Control specific**  **genotypic fraction** | **Common**  **genotypic fraction** | **Sum of combination of genotypes** |
| --- | --- | --- | --- | --- |
| ***2345** | **8** | **3** | **9** | **20** |
| **12*45** | **9** | **1** | **10** | **20** |
| **123*5** | **5** | **5** | **14** | **24** |
| **1*345** | **11** | **3** | **13** | **27** |
| **1234*** | **10** | **3** | **12** | **25** |
| **12345** | **14** | **7** | **14** | **35** |

**Table S4.B. Frequency of combination of genotypes** obtained after omission of one SNP from oral cancer, leukoplakia and control population

| **SNP combination** | **Control specific genotype** | **Leukoplakia specific genotype** | **Cancer specific genotype** | **Control- Leukoplakia genotypes** | **Control- Cancer genotypes** | **Cancer- Leukoplakia genotypes** | **Control Cancer-leukoplakia genotypes** | **Sum of genotypes** |
| --- | --- | --- | --- | --- | --- | --- | --- | --- |
| ***2345** | **6** | **5** | **8** | **4** | **4** | **3** | **36** | **66** |
| **1*345** | **6** | **5** | **2** | **4** | **8** | **4** | **34** | **63** |
| **12*45** | **5** | **6** | **5** | **5** | **6** | **2** | **41** | **70** |
| **123*5** | **2** | **6** | **7** | **6** | **5** | **4** | **34** | **64** |
| **1234*** | **7** | **5** | **7** | **6** | **7** | **3** | **33** | **68** |
| **12345** | **18** | **18** | **18** | **13** | **16** | **6** | **53** | **142** |
|  |  |  |  |  |  |  |  |  |
|  |  |  |  |  |  |  |  |  |
|  |  |  |  |  |  |  |  |  |
|  |  |  |  |  |  |  |  |  |
|  |  |  |  |  |  |  |  |  |
|  |  |  |  |  |  |  |  |  |
|  |  |  |  |  |  |  |  |  |

**Omitted SNPs are marked as ‘*’**
